# Pulmonary tuberculosis in outpatients in Sabah, Malaysia: advanced disease but low incidence of HIV co-infection

**DOI:** 10.1186/s12879-015-0758-6

**Published:** 2015-01-31

**Authors:** Timothy William, Uma Parameswaran, Wai Khew Lee, Tsin Wen Yeo, Nicholas M Anstey, Anna P Ralph

**Affiliations:** Infectious Diseases Unit, Queen Elizabeth Hospital, Kota Kinabalu, Sabah Malaysia; Infectious Diseases Society Sabah-Menzies School of Health Research Clinical Research Unit, Kota Kinabalu, Sabah Malaysia; Sabah Department of Health, Kota Kinabalu, Sabah Malaysia; Luyang Outpatient Clinic, Kota Kinabalu, Sabah Malaysia; Global and Tropical Health Division, Menzies School of Health Research, Darwin, Northern Territory Australia; Department of Medicine, Royal Darwin Hospital, Northern Territory, Darwin, Australia

## Abstract

**Background:**

Tuberculosis (TB) is generally well controlled in Malaysia, but remains an important problem in the nation’s eastern states. In order to better understand factors contributing to high TB rates in the eastern state of Sabah, our aims were to describe characteristics of patients with TB at a large outpatient clinic, and determine the prevalence of HIV co-infection. Additionally, we sought to test sensitivity and specificity of the locally-available point-of-care HIV test kits.

**Methods:**

We enrolled consenting adults with smear-positive pulmonary TB for a 2-year period at Luyang Clinic, Kota Kinabalu, Malaysia. Participants were questioned about ethnicity, smoking, prior TB, disease duration, symptoms and comorbidities. Chest radiographs were scored using a previously devised tool. HIV was tested after counselling using 2 point-of-care tests for each patient: the test routinely in use at the TB clinic (either Advanced Quality™ Rapid Anti-HIV 1&2, FACTS anti-HIV 1/2 RAPID or HIV (1 + 2) Antibody Colloidal Gold), and a comparator test (Abbott Determine™ HIV-1/2, Inverness Medical). Positive tests were confirmed by enzyme immunoassay (EIA), particle agglutination and line immunoassay.

**Results:**

176 participants were enrolled; 59 (33.5%) were non-Malaysians and 104 (59.1%) were male. Smoking rates were high (81/104 males, 77.9%), most had cavitary disease (51/145, 64.8%), and 81/176 (46.0%) had haemoptysis. The median period of symptoms prior to treatment onset was 8 weeks. Diabetes was present in 12. People with diabetes or other comorbidities had less severe TB, suggesting different healthcare seeking behaviours in this group. All participants consented to HIV testing: three (1.7%) were positive according to Determine™ and EIA, but one of these tested negative on the point-of-care test available at the clinic (Advanced Quality™ Rapid Anti-HIV 1&2). The low number of positive tests and changes in locally-available test type meant that accurate estimates of sensitivity and specificity were not possible.

**Conclusion:**

Patients had advanced disease at diagnosis, long diagnostic delays, low HIV co-infection rates, high smoking rates among males, and migrants may be over-represented. These findings provide important insights to guide local TB control efforts. Caution is required in using some point-of-care HIV tests, and ongoing quality control measures are of major importance.

## Background

Tuberculosis (TB) is a disease of major public health significance in Malaysia, with a current estimated incidence of 80 new cases annually per 100,000 population [1]. While important successes have been achieved in TB control nationwide [1,2], TB control has proven to be more challenging in Malaysian Borneo. The state of Sabah in eastern Malaysia on the island of Borneo contains approximately 10% of the country’s total population, but a disproportionally high burden of the country’s TB cases, estimated at 20-30% [3,4]. The case notification rate for Sabah has been sustained at higher levels of 144 to 217 per 100,000 population during recent decades while rates have fallen in other parts of Malaysia [5].

Reasons for the greater difficulty in achieving TB control in Sabah are unclear. The extent to which the local epidemic might be driven by HIV, migration from regional high-burden settings, drug resistance, delayed diagnosis, high smoking rates, diabetes or other factors, has not been clearly elucidated. Our own data indicate that drug resistance in the outpatient setting is uncommon (publication pending); this is supported by data from the local laboratory [4]. Nations which are close neighbours of Sabah have higher reported TB incidence than Malaysia (Philippines: 265/100,000; Indonesia 185/100,000 [1]), but the extent to which immigrants contribute to local TB case notifications is unclear. Migrants are thought to comprise approximately 27.8% of the Sabah population [6]. Regarding HIV as a contributor to TB rates, the rate of HIV co-infection in people with TB appears relatively low by international standards for Malaysia as a whole at 6.1% [1], with HIV status established in a high proportion of TB patients (97%) [1]. Rates of HIV among TB patients have not been published from Eastern Malaysia, but were found to be 7.7% in a 2012 study of 530 people in Western Malaysian hospitals [7]. Our unpublished data from the largest hospital in Sabah show an average of 63 new diagnoses of TB-HIV co-infection and 15 deaths annually between 2010 and 2013. Therefore although we recognise TB-HIV co-infection as a serious problem in the hospital setting, accurate data on TB-HIV co-infection in Sabah are lacking.

HIV point-of-care (POC) blood tests have been an essential and cost-effective component of HIV care in outpatient clinics, especially in resource-limited settings [8] but increasingly also in higher income settings [9]. A large number of manufacturers provide low-cost HIV POC tests. Such tests usually have reported sensitivity of >95% and specificity >99% under laboratory conditions [10]. The World Health Organisation (WHO) recommends a minimum HIV test sensitivity of 99% and specificity of 98% [11]. Problems with POC tests can exist however, including inadequate sensitivity [12], inadequate specificity [13,14] and faulty batches [15]. The simplicity of the tests also can provide a false sense of assurance about requirements for quality control measures and staff training. Lowest-cost tests can be especially appealing to budget-conscious decision makers but may underperform.

In order to better understand the factors contributing to high ongoing TB rates in Sabah, our aims were to determine epidemiological characteristics and HIV status of patients with tuberculosis at a large outpatient clinic in Kota Kinabalu Sabah. Additionally, we sought to test the sensitivity and specificity of the HIV POC test kit stocked by the TB clinic against another used-widely POC test. In the absence of readily-available onsite mycobacterial culture, we restricted the study to smear-positive pulmonary TB patients is order to include only those most likely to have true TB.

## Methods

### Setting

We enrolled participants at the Luyang Tuberculosis Outpatient Clinic, Kota Kinabalu, Malaysia, from July 4^th^ 2012 until July 3^rd^ 2014. This is the largest TB clinic in Kota Kinabalu treating almost 200 patients annually. It offers smear microscopy, directly observed therapy for TB, and receives referrals of suspected or confirmed TB cases from other Kota Kinabalu practitioners. Kota Kinabalu has a population of approximately 452,940 [6].

### Participants and procedures

Participants were eligible if they had smear-positive pulmonary TB, were aged ≥15 years, and provided written informed consent. They were asked about their ethnicity; smoking status including number of cigarettes per day and among ex-smokers, when they had quit; prior TB; disease duration and symptoms; and comorbidities. Comorbidity data were based on patient report rather than active screening. Ethnicity was determined by asking people what ethnicity they identified as, in which country they were born, and their current address. Ethnicity alone does not clearly define nationality due to the representation of the same ethnic groups (such as Bajau) in both Malaysia and the Philippines, and because immigrants may state their ethnicity as being that of their Malaysian spouse. TB care is provided free of charge to non-citizens.

TB disease severity was assessed using maximum recorded smear microscopy grade (1+, 2+ or 3+), presence of cavitary disease, and radiological score [16].

HIV pre-test counselling was provided both by the TB clinic doctor responsible for diagnosing TB and commencing treatment, and by the research nurse before collecting a blood sample. HIV post-test counselling was provided by the clinic doctor. Two HIV tests were performed in parallel for each patient, comprising a locally-available POC blood test provided for routine use at the clinic, and a comparator POC blood test provided by the study. The clinic-provided test types included: ‘Advanced Quality™ Rapid Anti-HIV 1&2’ (InTec Products, Inc, Xiamen, China, reported sensitivity and specificity 100% [17]), ‘FACTS anti-HIV 1/2 RAPID’ (Scientifacts Co Ltd, Malaysia, no reported sensitivity or specificity available) and ‘HIV (1 + 2) Antibody Colloidal Gold’ (KHB Shanghai Kehua Bioengineering Co PtyLtd, China, reported sensitivity and specificity 100% [18]). The comparator study test throughout the study was Abbott Determine™ HIV-1/2 (Inverness Medical, reported specificity 99.6%, sensitivity 99.4% [19]). Test kits were stored in air conditioned laboratory when not in use. New positive tests were confirmed by enzyme immunoassay (EIA), particle agglutination (PA) and line immunoassay (LIA) performed at Queen Elizabeth Hospital laboratory.

### Ethics

Ethical approval was obtained from the Medical Research Sub-Committee, Malaysian Ministry of Health (NMRR-11-1051-10491) and the Human Research Ethics Committee of Menzies School of Health Research, Australia (2010–1398).

### Statistics

Data were analysed using Stata™ 13.1. (Stata Corp, College Station, Texas, USA). Continuous variables were compared using Student’s T test or Wilcoxon Rank Sum test. Log transformation was applied to log-normal data (illness duration). Categorical variables were compared using Chi-squared or Fisher’s Exact test, as appropriate. Associations between patient characteristics and TB disease severity measures were tested using multivariable linear or logistic regression models. Logistic regression models were used to calculate odds ratios (OR) for associations between comorbidities and body mass index (BMI).

## Results

During the 2-year study period from July 4^th^ 2012 to July 3^rd^ 2014, 176 consenting participants were enrolled out of 395 people referred. 79 were ineligible, 12 were considered unsuitable by the research staff (e.g. too unwell or pregnant) and 128 did not consent. Chest x-rays were available for review in 145 people.

### Demographics

**Figure 1:**
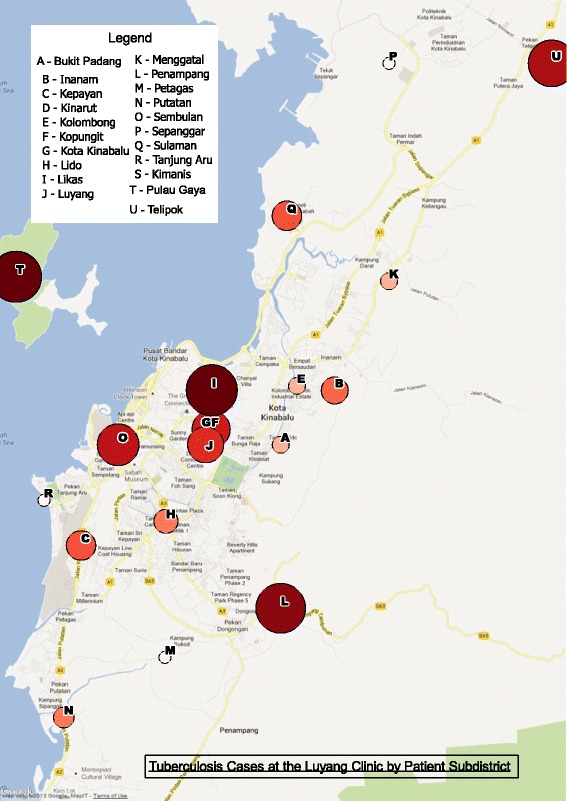
**Map of Kota Kinabalu demonstrating the geographical distribution of cases enrolled in the study.** Circles represent the proportional number of cases in each subdistrict, according to home address details provided by study participants.

Patient characteristics are shown in Table 1. Males were over-represented, and one third of patients (33.5%) were migrants. The geographical distribution of TB cases as determined from participants’ addresses is in shown in Figure 1. Some apparent hotspots appeared evident including Pulau Gaya, a migrant community off-shore island with an estimated population of 6000, connected to Kota Kinabalu via a ferry service.

**Table 1:** **Study participant characteristics**

**Characteristic**	**Number**
**Number**	176
**Age in years: median (range)**	30 (15–73)
**Male: no. (%)**	104 (59.1%)
**Nationality: no. (%)**	
Malaysian	117 (66.5%)*
*Indigenous*	106 (60.2%)
*Chinese*	10 (5.7%)
*Indian*	1 (0.6%)
Filipino	53 (30.1%)
Indonesian	6 (3.4%)
**Co-morbidities: no. (%)**	
Diabetes	12 (6.8%)
Hypertension	9 (5.1%)
Asthma	6 (3.4%)
HIV	3 (1.7%)
Hepatitis B	2 (1.1%)
Chronic renal disease	1 (0.6%)
Cardiovascular disease	1 (0.6%)
**Smoking status:**	
Current smoker	40 (22.7%)
Ex-smoker	50 (28.4%)
Non-smoker	86 (48.9%)
When smoking ceased: median no. months ago (IQR)	3.5 (2–24)
**Past TB**	13 (7.4%)
**Radiological severity:**	
Cavitary disease	51/145 (64.8%)
Score: mean (standard deviation)	69.4 (34.9)
**Smear grade: no. (%)**	
Scanty	27 (17.0%)
1+	46 (28.9%)
2+	40 (25.2%)
3+	46 (28.9%)
**BMI (kg/m2): mean (range)** ^**†**^	18.1 (10.0-31.1)
**Haemoptysis: no. (%)**	81 (46.0%)
**Reported symptom duration in weeks: median (range)** ^**†**^	8 (1–52)

*Percentages may not add up to 100 due to rounding.

^†^n = 174, data missing for 2 individuals.

### Participant characteristics and comorbidities

**Figure 2:**
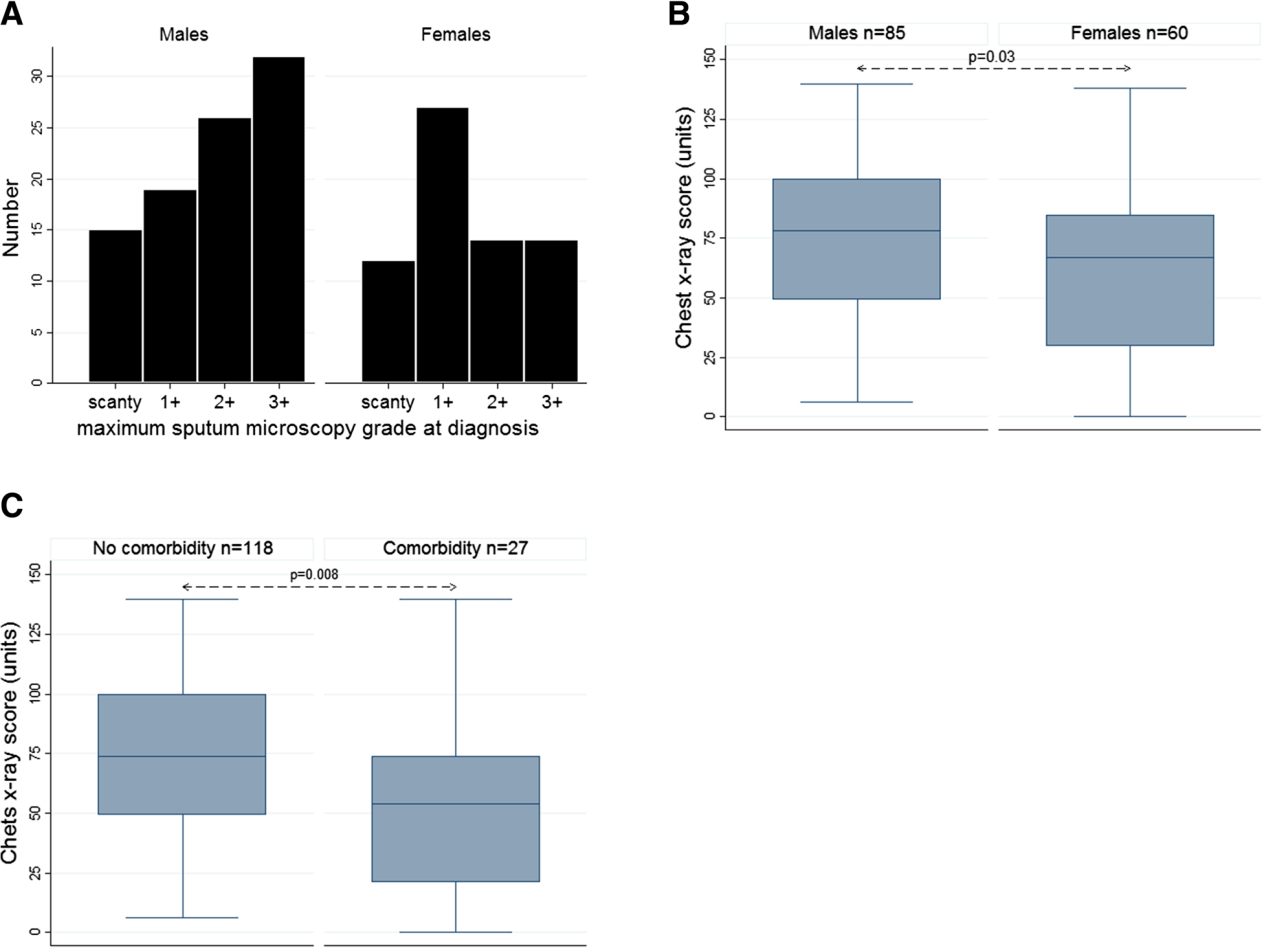
**Severity of pulmonary tuberculosis according to sex and presence or absence of non-HIV comorbidities. A**. Sputum microscopy grade in males and females. **B**. Baseline chest x-ray score in males and females. **C**. Baseline chest x-ray score in the presence and absence of recognised co-morbidities. Chest x-rays were available in 145 people. Any comorbidity included diabetes, asthma, hypertension, hepatitis B, chronic renal disease, cardiovascular disease, gastrointestinal disorder or chronic back pain. Box plots show 50th centile (line), 25th and 75th centiles (box) and minimum and maximum values (whiskers).

Patients had advanced disease at diagnosis on the whole, on the basis of low mean BMI, proportion with haemoptysis and proportion with cavitary disease (Table 1). Males tended to have more severe disease than females (Figure 2A and B). No statistically significant associations were found between ethnicity and TB severity, although slightly more people of non-Malaysian ethnicity had cavitary disease, 36/51 (70.6%) versus 58/94 (61.7%) in Malaysians (p = 0.285).

Regarding diagnostic delays, the median reported duration of symptoms prior to starting treatment was 8 weeks. Longer illness durations were observed in people with cavitary disease, high smear grade, and in the 6 individuals from Indonesia, but the latter was not statistically significant. Comparing geometric means of illness duration, these were: cavitary disease - 7.5 weeks, compared with 5.7 weeks in non-cavitary disease, p = 0.038; 3+ smear grade - 8.0 weeks, compared with 5.8 weeks in those <3+, p = 0.020; Indonesians - 10.3 weeks, compared with 6.1 weeks in Malaysians, p = 0.123. Illness duration was not associated with sex, age, BMI or presence of haemoptysis.

More than half the participants were current or ex-smokers. Nine females (12.5%) had ever smoked, versus 81 males (77.9%), p < 0.001. Among ex-smokers, the median time of cessation was 3.5 months prior to commencement of TB treatment.

Diabetes was the most commonly reported comorbidity, followed by hypertension and asthma (Table 1). Hepatitis B virus positivity, not routinely tested during most of the study, was recorded in 2 individuals. As expected, diabetes and hypertension were significantly associated with BMI and age (p ≤ 0.004 in each instance) Even controlling for BMI, the presence of diabetes, asthma or ‘any comorbidity’ (diabetes, asthma, hypertension, hepatitis B, chronic renal disease, cardiovascular disease, gastrointestinal disorder or chronic back pain but excluding HIV) was associated with *less* severe TB on X-ray (Figure 2C).

### HIV results

All participants consented to HIV testing. The HIV POC test stocked by the clinic changed regularly during the study period, with occasional stock-out periods, and also two instances in which Determine™ was unavailable (Table 2). Of the 176 participants analysed, 3 (1.7%) were HIV positive on Determine™ HIV-1/2 test (Table 2). Two were new diagnoses, both confirmed positive on EIA, PA and LIA. The third was a known pre-existing diagnosis. The locally-available test available in two instances (‘Advanced Quality™ Rapid Anti-HIV 1&2’) was positive in one participant but negative in the other. Although numbers were too small for definitive assessment, compared with Determine™, this provided a sensitivity of 50% and specificity of 100% for ‘Advanced Quality™ Rapid Anti-HIV 1&2’, in contrast with the information provided in the package insert [17]. The locally-available test used in the third instance (‘HIV (1 + 2) Antibody Colloidal Gold’) gave a positive result. Clinical summaries of the TB-HIV co-infected individuals are provided in Table 3. One died 4 months after the TB diagnosis, attributed to presumptive cerebral toxoplasmosis, with a differential diagnosis of cerebral tuberculoma.

**Table 2:** **HIV test results among smear-positive pulmonary TB patients, Luyang Clinic, 2012-14**

	**Determine™ result**			**TOTAL**
	**Positive**	**Negative**	**Not available**	
FACTS anti-HIV 1/2 RAPID (Scientifacts Co Ltd, Malaysia)	0	15	0	
Advanced Quality™ Rapid Anti-HIV 1&2, (InTec Products, Inc, Xiamen, China)	2	95	2	
HIV (1 + 2) Antibody Colloidal Gold (KHB Shanghai Kehua Bioengineering Co PtyLtd, China	1	31	0	
Not available	0	30	0	
**TOTAL**	**3**	**171**	**2**	**176**

**Table 3:** **Characteristics and outcomes of study participants with TB-HIV co-infection**

**Study participant**	**New HIV diagnosis made at time of TB diagnosis**	**CD4 T cell count: cell/μL**	**Clinical details**
57 year old Indigenous Malaysian male	Yes	328	Commenced on AZT, 3TC and efavirenz. TB cured.
22 year old Filipino female	No (diagnosed 1 year prior)	123	Migrant not eligible for free HIV treatment who had previously defaulted from antiretroviral therapy; remains under follow up with a view to re-start antiretrovirals if possible.
19 year old Indigenous Malaysian male	Yes	1	Attended HIV clinic for baseline assessment but did not return for results or commencement of treatment. Died in hospital with cerebral mass lesion 4 months after TB-HIV diagnosis, attributed to presumptive cerebral toxoplasmosis, with a differential diagnosis of cerebral tuberculoma.

## Discussion

We have found that TB patients in Kota Kinabalu have advanced disease at diagnosis, long diagnostic delays, low HIV co-infection rates, high smoking rates among males, and that migrants may be over-represented. These findings provide important insights to guide local TB control efforts. Although Malaysia is an upper middle-income country which has achieved impressive TB control at a national level, our study participants had severity measures similar to or worse than we have previously found in comparable patients in a low-resource Asian setting [20]. Forty six percent of participants in the current analysis had haemoptysis, nearly a third had a microscopy grade of 3+, and two thirds had cavitary disease. Although recall bias may influence people’s reported symptom duration, the median reported diagnostic delay of 8 weeks is 4 weeks longer than has been reported on average from elsewhere [21,22], and was associated with worse disease.

The presence of advanced disease suggests that earlier detection of pulmonary TB must become a priority for local healthcare services. This could be achieved through active case detection efforts, improved contact tracing, and a lower clinical threshold for considering TB, especially targeted at apparent geographical hotspots or at-risk communities. Mathematical modelling has indicated that control efforts targeting TB hotspots can be an efficient strategy to reduce wider TB rates [23]. Improved TB control among the migrant population group would have major individual and public health benefits, and impact favourably on Malaysian national TB statistics. Other Malaysian studies have estimated the proportion of cases occurring in migrants as being substantially fewer, at 10% in a 2004 study [2] and 17% in a study from 2000–2007 [24]. It makes sense that as TB control in Malaysia continues to improve, an increasing proportion of total TB cases will occur in people from neighbouring high-burden settings. In Australia for example, >85% of all TB cases now occur in migrants or visitors from high-burden countries [25].

Implementation of public policy to reduce smoking rates is greatly needed. Smoking increases the risk of TB infection and disease [26,27]. In this study, 51.1% of participants were current or ex-smokers, with many of the ex-smokers having quit only since the onset of respiratory symptoms. By comparison, in a recent study from Mexico, 24.4% of pulmonary TB patients were current smokers or smokers within the last 12 months [27]. Policy changes such as increased cigarette prices [28] are needed in Malaysia and other Asian nations in which smoking consumption rates remain very high among men, and are increasing among women [29]. We found that some patients had quit smoking due to pulmonary irritability (i.e. cough), sometimes at a date slightly before the time that they recalled the onset of TB symptoms. This raises the question of whether smoking cessation due to respiratory symptoms could be an early sign of tuberculosis in a high-burden setting, and hence whether smoking cessation due to increasing respiratory symptoms should prompt investigation for pulmonary TB.

Our data indicate that caution is required in the use of low-cost point-of-care HIV tests, which should be carefully evaluated before they become adopted as national standard of care. The low number of HIV positive results meant that test characteristics could not be ascertained with confidence. However, one instance of HIV positivity according to Determine™/EIA was negative on the clinic-provided test, raising major concerns. We had intended to test the sensitivity and specificity of one POC against Determine™ but the test stocked by the clinic changed throughout the study period, depending on clinic purchasing patterns which we could not influence. Our experience with low-cost rapid tests in clinical practice in Kota Kinabalu has included other instances of inadequate sensitivity, including in antenatal screening. We therefore recommend discontinuation of use of low-performing tests. A limitation of this study was that we did not use a gold standard comparator HIV test, although positive results were cross-checked with methods considered as gold standards. A study in Uganda has identified problems with low specificity (false positive results) with the Determine™ rapid tests [14], which we did not observe. One reason for lower performance of POC tests in field settings compared with manufacturer-provided laboratory data may be the temperature at which tests are often stored and used. Routine practice at our study site was for the clinic’s test kits belonging to be kept in the general clinical area which is not air conditioned, where the ambient temperature is often ≥25°C. A transition away from the traditional testing location of the laboratory to point-of-care testing in the clinic must be accompanied by training of clinical staff in the principles of laboratory quality control procedures.

It is welcome news that high ascertainment of HIV status was achieved, and that the prevalence of HIV co-infection among smear-positive TB patients in the outpatient setting was low at 1.7%. Comparison can be drawn with nearby eastern Indonesia (Papua Province), where the HIV rate among TB smear-positive outpatients is 13% [30]. However, one TB-HIV co-infected patient died, and barriers to institution of antiretroviral treatment were faced in another. Barriers are well described regionally [30] and globally [31]. Despite the reassuringly low community HIV rate we found, our clinical experience in the hospital setting in Kota Kinabalu is, contrastingly, of a major and rising problem with HIV and TB-HIV co-infection. Unpublished 2013 data from our Queen Elizabeth Hospital clinic, the main referral centre for HIV management in Kota Kinabalu, show 253 new HIV diagnoses. Seventy seven had TB co-infection, and 12 died in 2013. Thus there is no scope for complacency.

Limitations of this study are that participants may not be representative of Malaysian Borneo TB patients as a whole. Males were over-represented, but male over-representation among PTB patients is not uncommon in Asia [32], for a variety of reasons reviewed elsewhere [33]. Our selection criteria (smear-positive outpatients) would have selected for a group less likely to have HIV. Reluctance to have an HIV test was not cited as a reason for non-consent – people not enrolled in the study also had HIV testing performed as part of routine clinical care. Non-HIV co-morbidity data were based on patient report only; hence co-morbidities were likely to be under-estimated. Determination of true ethnicity can be hampered by a reluctance to admit to non-local status, partly related to fears about health care costs for non- residents, or deportation; we reassured people that TB treatment is free regardless of ethnicity, and ethnicity would remain confidential; also, we did not rely on stated ethnicity alone, as described in the Methods.

A somewhat unexpected finding was that people with co-morbidities had less-severe disease. This might be because people knowledgeable of their co-morbidities may be those more likely to seek health care earlier, and to be engaged in health care services. Just as TB is an important opportunity to detect HIV, so too is it an important opportunity to detect diabetes. Screening for diabetes could be an important additional strategy to add to TB care in Kota Kinabalu, particularly in older individuals with higher BMI. The finding of more advanced disease in males might also have been related to their health-seeking behaviour; alternatively their high smoking rates may have contributed, although we could not find a statistically significant association between smoking and severity per se.

## Conclusions

In summary, the low rates of TB-HIV co-infection we identified provide optimism for local TB control. We recommend that careful quality control procedures be applied to HIV point-of-care testing, and that purchasing of test kits be guided by evidence of adequate test characteristics in keeping with WHO recommendations. Our findings have prompted changed local practices regarding HIV test kit use, and improved communication between the TB and HIV clinics. We suggest that a cost effective strategy for controlling local TB rates would be a greater focus on geographical TB foci comprising socioeconomically disadvantaged migrant populations in Sabah. Since TB rates in eastern Malaysia have a disproportionate influence on national rates, successes in Sabah could translate into a fall in national TB rates. Local operational research in strengthening TB control is required.

## Abbreviations

BMI: Body mass index
EIA: Enzyme immunoassay
HIV: Human immunodeficiency virus
LIA: Line immuoassay
TB: Tuberculosis
OR: Odds ratio
PA: Particle agglutination
POC: Point of care
